# A novel prognostic model based on Golgi apparatus-related genes predicts patient survival in glioblastoma

**DOI:** 10.1515/med-2026-1479

**Published:** 2026-09-08

**Authors:** Hongliu Wang, Zhigang Liao, Lihua Jin, Youkun Dong, Hanjiang Cui, Xihong Xing, Feng Lu, Kedong Jiang, Xiaoli Hu, Xiaofen Bo, Li Ni

**Affiliations:** Department of Neurosurgery, The Second People’s Hospital of Jing Zhou & The Affiliated Hospital of Hubei College of Chinese Medicine, Jing Zhou, Hubei Province, PR China; Department of Stomatology, The Central Hospital of Wuhan, Tongji Medical College, Huazhong University of Science and Technology Department of General Practice, Wuhan, Hubei Province, PR China; Department of General Practice, The Central Hospital of Wuhan, Tongji Medical College, Huazhong University of Science and Technology Department of General Practice, Wuhan, Hubei Province, PR China

**Keywords:** glioblastoma, Golgi apparatus-related genes, prognostic model, immune infiltration analysis, drug sensitivity

## Abstract

**Objectives:**

Glioblastoma (GBM) is a highly aggressive brain cancer. Golgi dysfunction plays a role in tumorigenesis.

**Methods:**

This study analyzed the role of Golgi apparatus-related genes (GARGs) in GBM using bioinformatics. Prognostic genes were identified using differential expression, Cox, and Lasso analyses. A prognostic model and nomogram were constructed based on gene expression and clinical characteristics. Immune infiltration and drug sensitivity analyses were performed in different risk groups. Reverse transcription quantitative PCR (RT-qPCR) was employed to explore the expression of prognostic genes.

**Results:**

A total of 5 prognostic genes (CD14, FMOD, LGR6, SLC16A13, and KDELR2) of GBM were identified, and on the basis of the prognostic genes, a prognostic model was built. There was a significant survival difference between patients in the high and low risk groups for GBM. Receiver operating characteristic (ROC) curve indicated that the predictive effect of the prognostic model in the training set was moderate, with an AUC value of around 0.7. The nomogram showed that risk score had a better predictive effect than age. Drug sensitivity analysis demonstrated that sorafenib was more sensitive in the low-risk group of GBM. RT-qPCR results revealed that compared with the control group, the expression levels of CD14 and SLC16A13 were significantly downregulated in GBM cell lines, while FMOD and LGR6 were markedly upregulated. The expression of KDELR2 exhibited obvious cell line-dependent variation.

**Conclusions:**

In summary, this study establishes a Golgi apparatus-related gene signature that not only offers prognostic insights but may also informs tailored therapeutic strategies for GBM patients.

## Introduction

Glioblastoma (GBM) represents the most prevalent and aggressive form of primary malignant brain tumor occurring in adults. It is characterized by rapid proliferation, widespread infiltration, and high resistance to anticancer drugs [1], 2]. Consequently, the prognosis for patients with GBM is bleak, with an average overall survival ranging from 10 to 20 months [3]. The main treatments for GBM include surgical resection, adjuvant radiotherapy, and chemotherapy [4]. Despite significant advancements in treatment strategies for GBM, it remains incurable due to its resistance to conventional therapy and postoperative metastasis and recurrence. Survival improvement seems to have plateaued, with recurrence posing the greatest clinical challenge [5], 6]. The complexity of the GBM immune microenvironment may impact clinical outcomes during disease progression [7]. Tumor progression involves various mechanisms and molecules [8]. Therefore, it is imperative to ascertain stable and dependable molecular signatures for accurately assessing the prognosis of GBM patients and devising more efficacious treatment strategies.

The Golgi apparatus is an essential organelle, serving as the central hub for intracellular material transport, synthesis, and modification. It plays a pivotal role in essential biological processes encompassing protein synthesis, modification, and localization, thereby exerting a crucial influence on cellular function and regulation [9], 10]. The cytoplasmic surface of the Golgi plays a crucial role in carrying and regulating the signaling cascade, particularly impacting the DNA damage response and mitosis [11]. Dysfunction of the Golgi may be implicated in cancer, infection, neurodegeneration, and cardiovascular disease [12]. Furthermore, the Golgi facilitates numerous cellular mechanisms involved in the development of cancer, including angiogenesis, the innate immune response, tumor invasion, and migration [13]. Because of the unique pathophysiological features and therapeutic challenges of GBM, understanding the complex relationship between the Golgi and GBM is of critical importance in identifying new therapeutic targets and enhancing patient prognosis. Artificial intelligence (AI), which mainly includes machine learning (ML) and deep learning (DL) computational approaches, has demonstrated considerable potential in the detection and diagnosis of various cancer types – from breast and lung to brain tumors – by leveraging imaging, genomic, and clinical data to enhance accuracy and early intervention [14], 15]. Studies show that Golgi apparatus-related genes (GARGs) typically exhibit mutations in tumors, which increases the risk of metastasis and poor prognosis, and have been used as cancer diagnostic biomarkers [12], 16]. However, while most AI-driven studies in GBM have focused on imaging or transcriptome-wide approaches [16], [17], [18], few have systematically linked specific organelle-related gene sets to patient prognosis. This gap motivates a more biologically informed, pathway-centric modeling strategy focused on the Golgi.

Notably, although GARG signatures have recently been explored in other malignancies such as osteosarcoma, gastric cancer, and breast cancer [19], [20], [21], comprehensive and methodologically rigorous research focusing on GARGs in GBM is still lacking. Most previous studies relied merely on differential expression and survival analyses, while ignoring rigorous statistical testing and biological consistency assessment for candidate genes. To address these limitations, the present study adopted a more rigorous screening process such as proportional risk (PH) hypothesis testing, aiming to construct a more robust and biologically meaningful prognostic model for GBM. We hypothesized that GARGs are closely involved in GBM pathogenesis and may serve as effective prognostic indicators. This study aimed to clarify whether Golgi function-related genes can be integrated to develop a robust prognostic signature for GBM. Findings from this work will advance the understanding of GARG-mediated mechanisms in GBM and provide potential molecular targets for clinical prognostic evaluation and therapeutic development.

## Materials and methods

### Data collection

Transcriptome data of The Cancer Genome Atlas-Glioblastoma (TCGA-GBM), corresponding clinical traits and subsequent information (including age, gender, and Karnofsky performance status [KPS] score), were retrieved and downloaded from the publicly accessible TCGA database (https://portal.gdc.cancer.gov/). There were 166 GBM samples and 5 normal samples, of which 165 GBM patients had survival information. It is important to note, however, that the TCGA-GBM cohort primarily comprises patients from large academic centers who were eligible for surgical intervention. This may introduce selection bias by underrepresenting those with poor performance status or inaccessible tumors, as well as sampling bias due to intratumoral heterogeneity. In addition, as GBM is associated with short median survival, potential overrepresentation of long-term survivors in the dataset may affect prognostic model estimation.

To mitigate these biases and enhance the generalizability of our findings, we supplemented the normal transcriptomic data with 1,148 samples from the genotype-tissue expression (GTEx) database. The combined TCGA-GBM and GTEx-normal datasets were utilized as the training set, totaling 166 GBM samples and 1,153 normal samples.

For external validation, two independent cohorts from the GEO database (https://www.ncbi.nlm.nih.gov/geo) were utilized to assess model generalizability. The GSE83300 dataset comprised 50 GBM samples, all of which contained > 50 % tumor cells as confirmed by microscopic evaluation. These specimens were collected from patients who underwent surgery between September 2005 and June 2009 and were used for expression profiling analysis. The GSE74187 dataset included 60 GBM samples obtained via surgical resection at Tian Tan Hospital from 2008 to 2010, with comprehensive overall survival (OS) and progression-free survival (PFS) records. Together, these validation sets enhance the robustness and clinical relevance of the prognostic model by evaluating its performance across heterogeneous patient populations and sampling conditions.

Furthermore, 1,653 GARGs were collected from the MsigDB database with the related entry “GOCC_GOLGI_APPARATUS”.

### Identification and functional enrichment analysis of differentially expressed GARGs (DE-GARGs)

To find differentially expressed genes (DEGs) in the training set, the R package “DESeq2” was used [22], and the screening parameters were |log_2_Fold Change (FC)|>1, p*<*0.05. Then the “ggplot2” and “ComplexHeatmap” R packages were utilized to plot volcano maps and heat maps respectively to show the situation of differential gene expression [23], 24]. Finally, DE-GARGs were obtained by intersecting the selected DEGs with GARGs. The R-package “clusterProfiler” was utilized for Gene Ontology (GO) and Kyoto Encyclopedia of Genes and Genomes (KEGG) functional enrichment of DE-GARGs [25].

### Chromosome localization analysis and establishment of protein-protein interaction (PPI) network

Chromosomal localization analysis of DE-GARGs was carried out using the “RCircos” R package [26]. Protein-protein interaction (PPI) networks for the DE-GARGs were constructed with the STRING database (http://string.embl.de/) (confidence score ≥ 0.4) and visualized in Cytoscape and cytoHubba plug-in.

### Gene mutation analysis and copy number variation (CNV) analysis

The “maftools” R package was utilized for the analysis of genetic mutations in the training set of GBM patients [27], with results visualized using a waterfall plot. The tumor mutational burden (TMB) score was calculated for each patient using the formula: TMB score=(total mutation/total covering base) × 10^6^. CNV data for model genes were downloaded from UCSC Xena (https://xenabrowser.net/datapages/) and the frequency of gain or loss in DE-GARGs was counted.

### Cox and Lasso analysis to screen prognostic genes

Using the R package “survival,” univariate Cox regression analysis was performed on the DE-GARGs of GBM samples in the training set [28]. Genes significantly associated with survival (hazard ratio [HR]≠1, p*<*0.05) were initially selected. These candidate genes were subsequently evaluated for the PH assumption test using the cox.zph function. Only genes satisfying the PH assumption (p>0.05) and exhibiting consistent directions in both hazard risk and differential expression were retained for subsequent analysis. A multivariate Cox analysis (HR≠1, p*<*0.05) was then performed and the genes were filtered using stepwise regression with the “step” R package [29]. Finally, Lasso-Cox regression analysis was carried out with the R package “glmnet” [30]. Prognostic genes were screened using 10-fold cross-validation, and the corresponding regression coefficients for each prognostic gene were obtained.

### Construction of a prognostic model and analysis of prognostic gene expression

A prognostic model for GBM was developed based on the expression profiles of previously identified prognostic genes within the training cohort. The risk score for each patient was calculated using the formula: $\text{risk score}=\overset{n}{\underset{i=0}{\sum }}*\beta i*\text{xi}$ (*βi* is the weight coefficient of each gene, xi is the expression level of each gene). This score was computed for all GBM patients across the three datasets (training set, GSE83300 dataset, and GSE74187 dataset). Patients were then ranked according to their risk scores from low to high, and risk score distributions were visualized. Overall survival analysis was performed utilizing the “Survival” R package. Time-dependent receiver operating characteristic (ROC) curves for 1-, 2-, and 3-year survival were generated with the “survivalROC” R package [31], and the corresponding area under the curve (AUC) values were computed. To dichotomize patients into high- and low-risk groups, the optimal risk score cut-off was determined with the surv_cutpoint function from the R package “survminer” (minpro*p=*0.3) [32]. Kaplan–Meier (KM) survival curves were subsequently plotted, and survival differences between the two groups were assessed using the log-rank test. Lastly, the expression patterns of the prognostic genes across the different risk categories were visualized using a heatmap.

### Gene set enrichment analysis (GSEA) and gene set variation analysis (GSVA)

Based on risk score and prognostic genes of GBM, Spearman correlation analysis was performed with all genes in the genome. The resulting correlation coefficients were used as the ranking metric for GSEA, which was conducted utilizing the “clusterProfiler” R package. Significantly enriched gene sets were identified with a threshold of p*<*0.05 and |NES|>1. The gene sets related to KEGG and Hallmark were obtained from MSigDB. The “GSVA” R package was used to determine GSVA scores for multiple pathways [33], and the “limma” package was employed to analyze the variances among all entries [34], with significance defined as |t|>2 and p*<*0.05.

### Construction of prognostic nomogram

The clinical characteristics of GBM patients (including age, gender, KPS) were obtained from the TCGA database. Each clinical variable was categorized, and boxplots were generated to visualize risk score distributions across subgroups. Univariate and multivariate Cox regression analyses were then performed on the training set, incorporating age, gender, KPS, and the risk score to identify independent prognostic factors. These factors were used to construct a nomogram predicting 1-, 2-, and 3-year overall survival of GBM patients. ROC curves, calibration curves, and DCA curves were plotted to validate the precision and clinical utility of the nomogram.

### Immune infiltration analysis and drug sensitivity analysis

The “estimate” R package was utilized to calculate immune score, stromal score, and estimate score in samples of high and low risk groups in the training set [35]. The relative abundances of 22 immune cell types in each GBM sample were quantified using CIBERSORT with the LM22 signature gene set (1,000 permutations). The resulting infiltration profiles were visualized as a stacked bar plot for both risk groups using the “ggplot2” R package. Wilcoxon test was subsequently applied to compare the proportions of each cell type between high- and low-risk groups, and the Pearson correlation between significantly differential immune cells and risk scores was further analyzed. The “oncopredict” R package was utilized to calculate the IC_50_ value of commonly used chemotherapy drugs [36].

### Reverse transcription quantitative PCR (RT-qPCR)

SVG P12 cells (Wuhan Shangen Biotechnology Co., Ltd.) and U87-MG cells (Xiamen Yimo Biotechnology Co., Ltd.) were cultured in DMEM medium, while T98G cells (Xiamen Yimo Biotechnology Co., Ltd.) were cultured in MEM medium. Both culture media were supplemented with 10 % fetal bovine serum (FBS, Gibco, USA) and 1 % penicillin-streptomycin (P/S, Gibco, USA). All cell lines were incubated in a humidified incubator at 37 °C with 5 % CO_2_ and used to detect the expression levels of prognostic-related genes.

Total RNA was extracted from each cell line, and the purity and concentration of RNA were determined using a NanoDrop-500 spectrophotometer. Subsequently, qualified RNA was reverse-transcribed into complementary DNA (cDNA) using ABScript III RT Master Mix for RT-qPCR with gDNA Remover (RK20429, Abconal, China). RT-qPCR was performed using Genious 2X SYBR Green Fast RT-qPCR Mix (RK21205, Abconal, China). The thermal cycling conditions were set as follows: initial denaturation at 95 °C for 3 min, followed by 40–45 cycles of 95 °C for 5 s and 60 °C for 30 s. The relative mRNA expression levels of target prognostic genes were calculated using the 2^−ΔΔCt^ method. The internal reference gene was the GAPDH. All primer sequences used in this study are listed in Table 1.

**Table 1:** The primer sequences of prognostic genes in RT-qPCR.

Name	Sequence 5′–3′
GAPDH	F:5′-CTGGGCTACACTGAGCACC-3′
	R:5′-AAGTGGTCGTTGAGGGCAATG-3′
CD14	F:5′-ACGCCAGAACCTTGTGAGC-3′
	R:5′-GCATGGATCTCCACCTCTACTG-3′
FMOD	F:5′-ATTGGTGGTTCCACTACCTCC-3′
	R:5′-GGTAAGGCTCGTAGGTCTCATA-3′
LGR6	F:5′-ACACAACCGCATCTGGGAAAT-3′
	R:5′-CGTTCCAGCTAAGATCCAGGG-3′
SLC16A13	F:5′-CTCTCAGCGTTCTTCCAGTCG-3′
	R:5′-GCTGCACCGCGATTCCTAT-3′
KDELR2	F:5′-GCACTGGTCTTCACAACTCGT-3′
	R:5′-AGATCAGGTACACTGTGGCATA-3′

### Statistical analysis

All analyses were performed in R software (version 4.4.1). The Wilcoxon test was used to compare continuous variables across groups, such as immune cell infiltration ratios, ESTIMATE scores, and drug sensitivity (IC50). Associations between significantly differential immune cells and risk scores were analyzed by calculating Pearson’s correlation coefficients. A p-value<0.05 was considered significant. Statistical significance is denoted by asterisks: ns, p>0.05, * for p<0.05, ** for p<0.01, *** for p<0.001, **** for p<0.0001.

## Results

### GO and KEGG functional enrichment analysis of DE-GARGs

**Figure 1:**
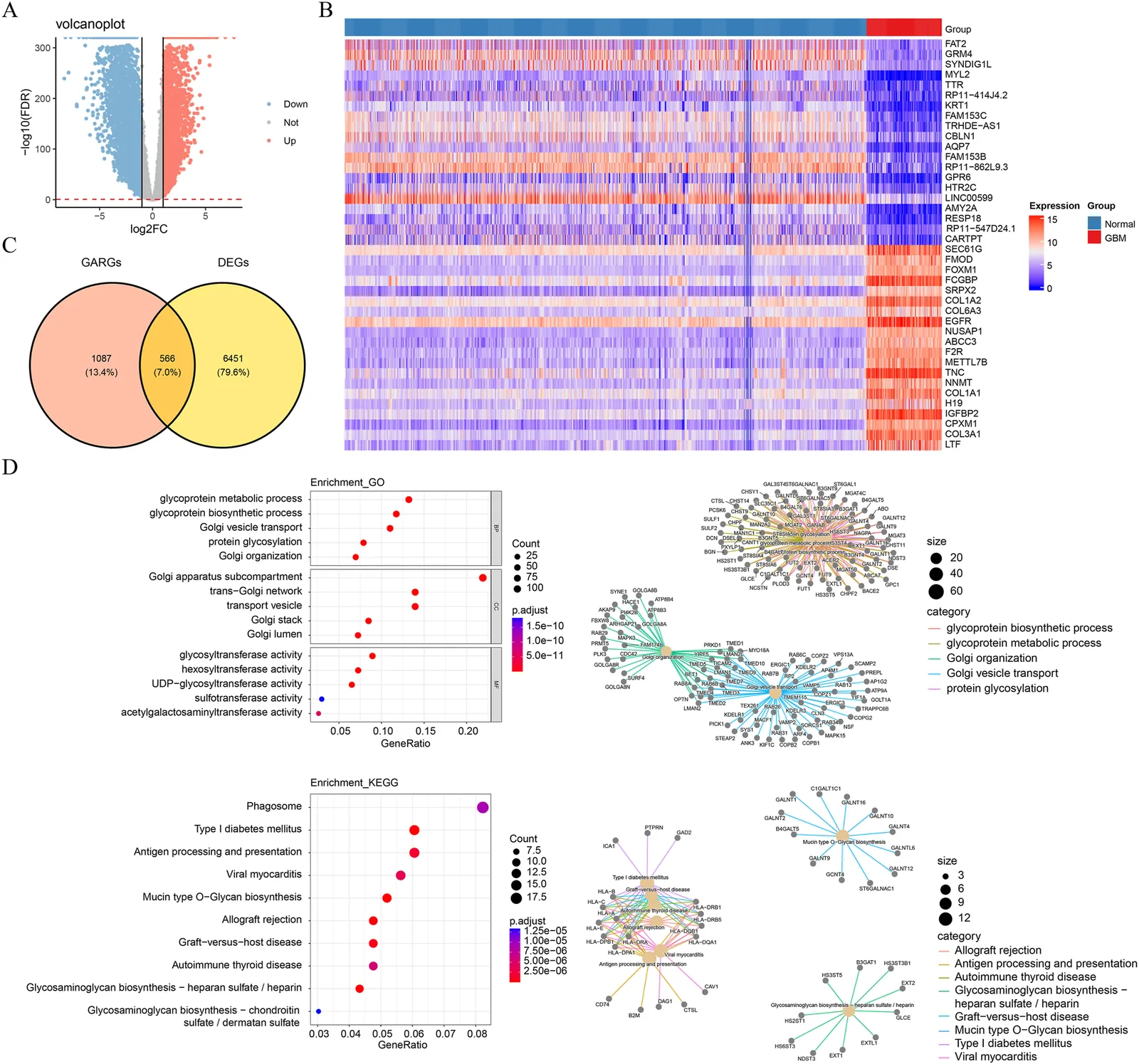
Gene ontology (GO) and kyoto encyclopedia of genes and genomes (KEGG) functional enrichment analysis of differentially expressed Golgi apparatus-related genes (DE-GARGs). (A) Volcano map of differentially expressed genes (DEGs). Red dots represented genes that were differentially up-regulated, blue dots represented genes that were differentially down-regulated, and grey dots represented genes that were not statistically significant. (B) Heat map of DEGs. The horizontal coordinate represented the sample, with blue and red above represented normal and GBM samples, respectively. The vertical coordinates represent genes, with highly expressed genes in red and lowly expressed genes in blue within. (C) Venn diagram for DE-GARGs. (D) functional enrichment analysis of DE-GARGs. The left side was the scatter plot of enrichment analysis, and the right side was the chord plot of enrichment analysis.

A total of 7,017 DEGs were screened in training set of GBM (|log_2_FC|>1, p*<*0.05). Compared with normal samples, there were 2,866 up-expressed genes and 4,151 down-expressed genes in GBM samples (Figure 1A, Supplementary Table 1). The top 20 up-expressed and down-expressed genes were shown in the heat map (Figure 1B). Then, the overlap between the 7,017 DEGs and 1,653 GARGs yielded 566 genes, which were defined as DE-GARGs (Figure 1C). GO enrichment analysis identified 1,211 items in the three modules, of which 959 items were significantly enriched in biological processes (BP), 133 items were significantly enriched in cell components (CC), and 119 items were significantly enriched in molecular functions (MF) (Supplementary Table 2). Additionally, each module’s top 5 pathways were displayed in order of p-value, from small to large, mainly including “glycoprotein metabolic process”, “glycoprotein biosynthetic process”, “Golgi vesicle transport, “Golgi organization,” and “protein glycosylation” (p<0.0001) (Figure 1D). Concurrently, the top 10 of 45 significant terms in KEGG pathway analysis were shown in Figure 1D, with the most notable being “Mucin type O-Glycan biosynthesis,” “Type I diabetes mellitus,” and “Glycosaminoglycan biosynthesis-heparan sulfate/heparin” (p<0.0001), among others (Supplementary Table 2).

### Chromosome localization analysis and PPI network construction of DE-GARGs

**Figure 2:**
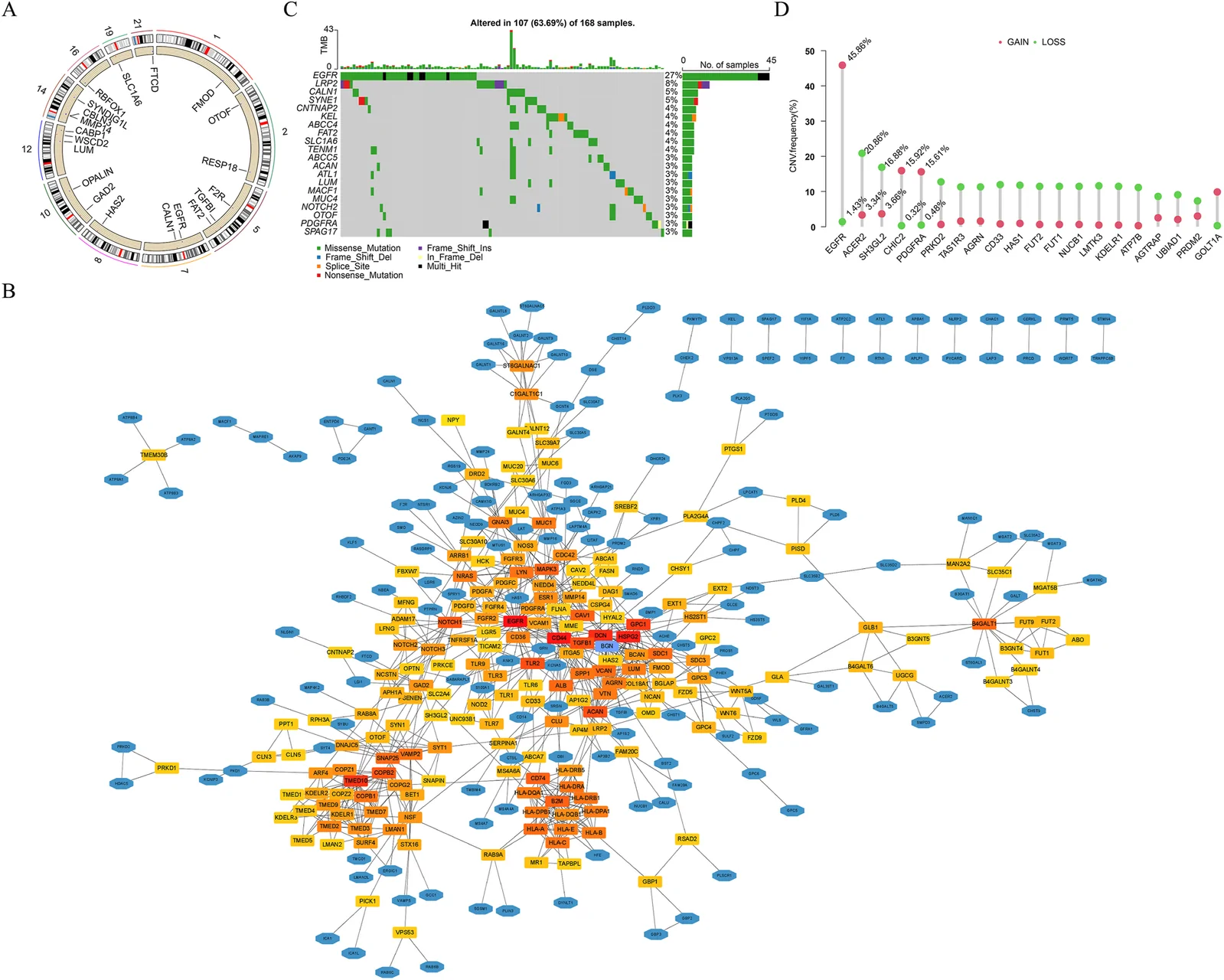
Chromosomal localisation analysis and mutation analysis of DE-GARGs. (A) Chromosomal localisation analysis of DE-GARGs, with different chromosome distributions in the outer ring, dots in the yellow circle indicate the expression of the relevant genes, and red represented log_2_Fold change (FC)>1 and blue represented log_2_FC<−1. (B) Protein-protein interaction (PPI) network map of DE-GARGs. Colours closer to red indicated greater connectivity. (C) The top 20 genes with the highest mutation frequency in GBM samples. The top of the figure showed the TMB scores for each sample, and the waterfall-like part in the middle showed the mutations of each gene in each sample, with each row being a gene and each column being a sample. (D) the top 20 genes with the highest copy number variation (CNV) frequencies in GBM samples.

To further understand the specific locations of DE-GARGs on chromosomes, we performed chromosomal localization analysis and visualized the locations of the top 20 genes with the highest log_2_FC changes on chromosomes. The results indicated that the majority of DE-GARGs were located on chromosomes 5, 12, and 14. (Figure 2A). The protein interaction network analysis of DE-GARGs showed that EGFR, CD44, DCN, HSPG2, TMED10 and GPC1 had high connectivity, which were highlighted in red (Figure 2B).

### The effect of gene mutation and CNV in DE-GARGs on the development of GBM

To characterize the genetic alterations in DE-GARGs, we first analyzed their mutation landscape. The waterfall plot displays the top 20 most frequently mutated genes, among which EGFR (28 %), LRP2 (8 %), and CALN1 (5 %) showed the highest mutation frequencies (Figure 2C). To further understand the CNV in DE-GARGs, we calculated the frequency of gain or loss in DE-GARGs, and identified the top 20 genes with the most frequent CNV alterations. Notably, EGFR also ranked highest in terms of CNV frequency of gain of 45.86 % vs. 1.43 % for loss (Figure 2D).

### Screening of prognostic genes for GBM

**Figure 3:**
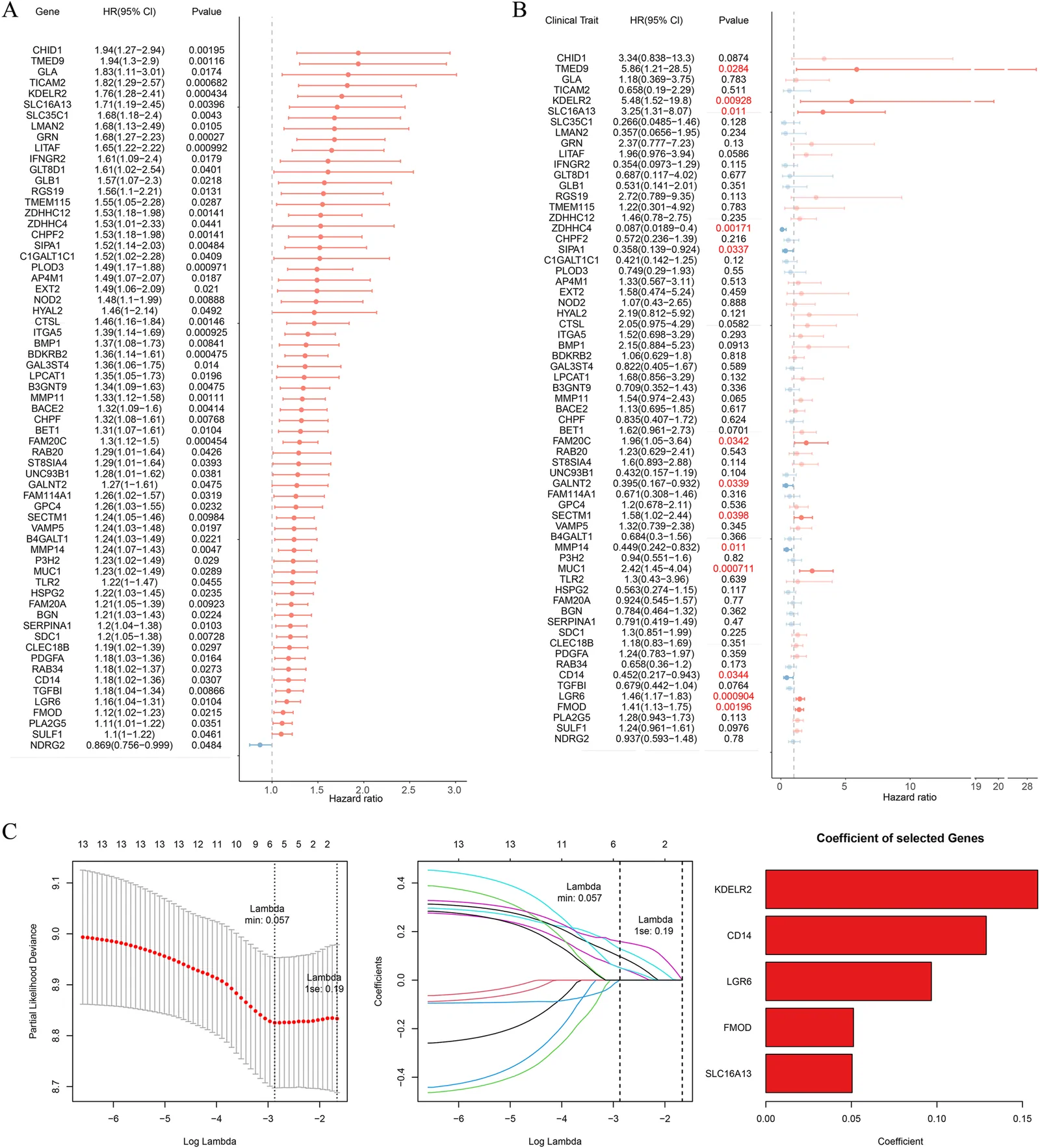
Screening of prognostic genes for GBM. (A) Univariate cox regression analysis of DE-GARGs. (B) Multivariate cox regression analysis of DE-GARGs. (C) Lasso regression analysis. On the left, the horizontal coordinate WSS log(Lambda), the vertical coordinate represented the error of cross-validation, and deviance represented the proportion of the residual interpreted by the model. In the middle figure, the horizontal coordinate was log(Lambda) and the vertical coordinate was the coefficient of the genes. The figure on the right showed the coefficients of prognostic genes.

To preliminarily screen genes associated with GBM survival, univariate Cox regression analysis was conducted on DE-GARGs, and finally 99 genes significantly associated with survival were screened (HR≠1, p*<*0.05) (Figure 3A). The PH assumption was verified for the univariate Cox models, and 94 genes satisfied this assumption (p>0.05). We then screened for genes showing consistent directionality in both hazard risk and differential expression, yielding 65 candidate genes (Supplementary Table 3). Following this, multivariate Cox analysis of these 65 candidates identified 13 significant genes (HR≠1, p*<*0.05) (Figure 3B). Subsequently, these 13 genes were analyzed using Lasso-Cox regression with 10-fold cross-validation to select the final prognostic genes. Finally, 5 prognostic genes and their corresponding coefficients were identified (Figure 3C, Supplementary Table 4), including CD14, FMOD, LGR6, SLC16A13, and KDELR2.

### Survival difference analysis between high and low risk groups of GBM

**Figure 4:**
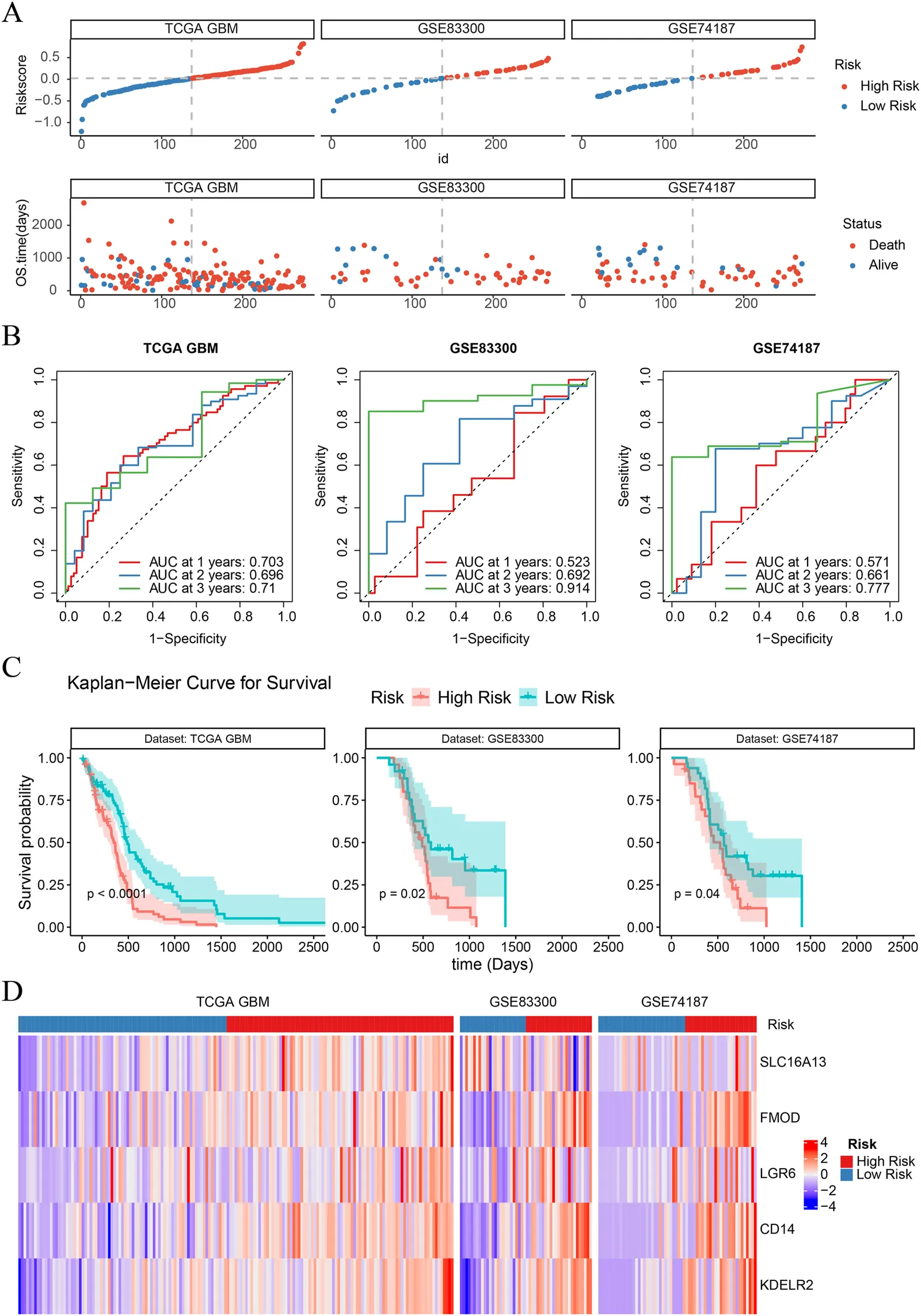
Survival difference analysis between high and low risk groups of GBM. (A) Risk curve analysis of high risk and low risk of GBM patients. (B) Receiver operating characteristic (ROC) curve analysis in TCGA-GBM, GSE83300 and GSE74187 datasets. The vertical coordinate is true positive, the horizontal coordinate is false positive, and the area below the curve in the figure was called AUC value. (C) Kaplan–Meier (KM) curve analysis of prognostic model. The horizontal coordinate represented time and the vertical coordinate represented survival probability. (D) heatmap analysis of expression for prognostic genes. Horizontal coordinated were different samples and vertical coordinates were prognostic genes.

The risk score for each GBM patient was calculated in the GBM training set, as well as in the GSE83300 and GSE74187 datasets, and created a risk curve that was plotted from low to high based on the risk score. The study revealed that mortality rates were significantly higher among the high-risk groups compared to the low-risk cohorts, indicative of notably lower survival prospects for individuals within the high-risk samples (Figure 4A). The performance of the prognostic model was further assessed across three independent datasets, with survival outcomes evaluated at 1-, 2-, and 3-year timepoints. Based on ROC curve analysis, the model achieved AUC values of approximately 0.7 at all three timepoints in the training set. Consistent results were observed in the GSE83300 and GSE74187 validation datasets, where the 2- and 3-year AUC values also remained around 0.7, indicating a stable, moderate discriminative ability of the model in predicting prognostic status (Figure 4B). The KM survival curve indicated that there were significant differences between GBM patients in the different groups in the three datasets (p*<*0.05). Patients belonging to high-risk groups exhibited significantly poorer survival rates in comparison to those in low-risk groups (Figure 4C). Finally, the expression levels of 5 prognostic genes were displayed, which 5 prognostic genes generally had expression levels in the high-risk group than in the low-risk group (Figure 4D). In conclusion, the prognostic model had the potential to accurately forecast the survival status of patients with GBM.

### Risk score as an independent prognostic factor of GBM

**Figure 5:**
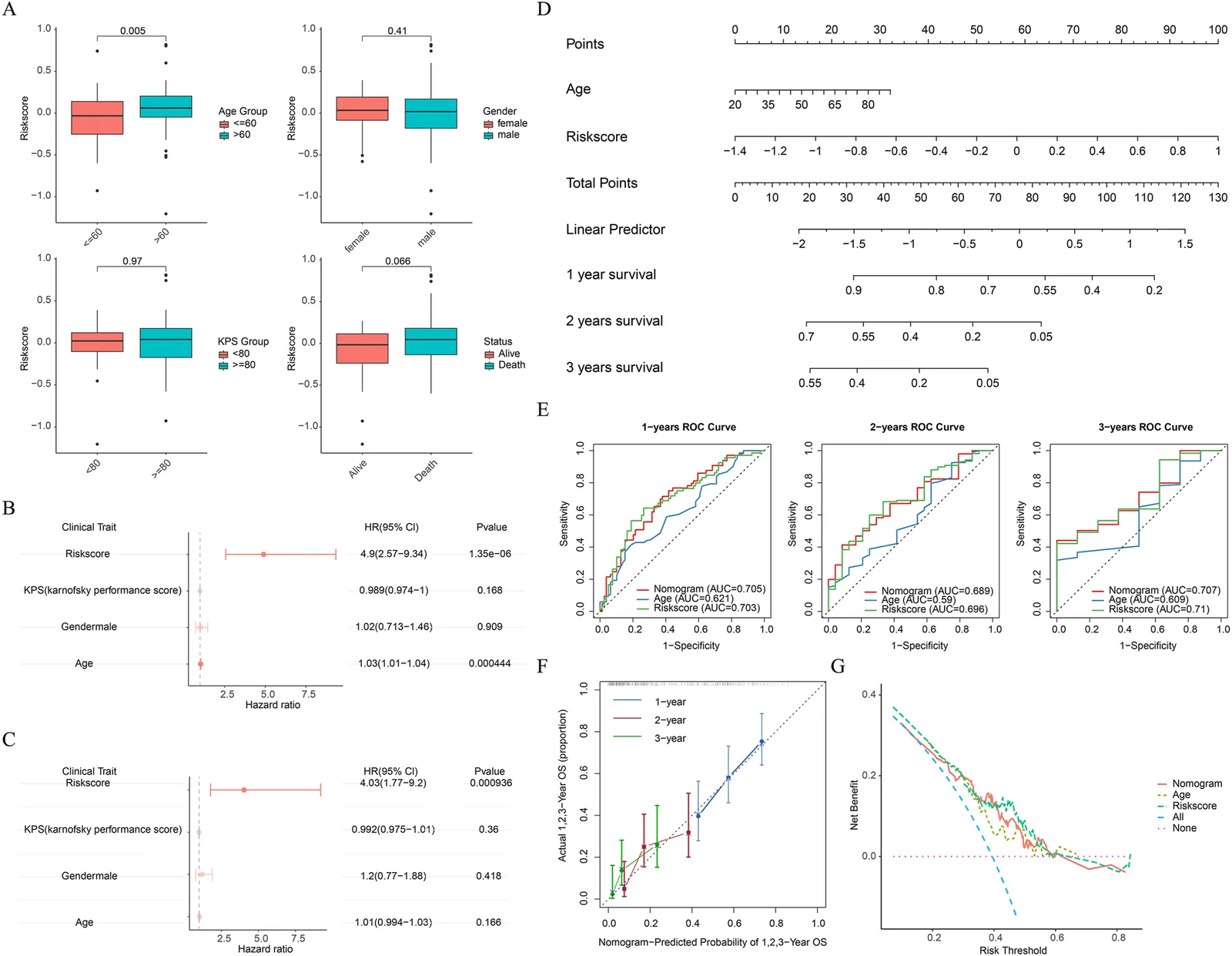
Correlation analysis between prognostic risk model and clinical features. (A) Box plots of risk score differences in different clinical feature subgroups. (B) Univariate cox analysis of clinical features and risk score. (C) Multivariate cox analysis of clinical features and risk score. (D) nomogram model analysis. (E) calibration curve analysis of nomogram. The horizontal coordinate was the predicted event incidence, and the vertical coordinate was the actual event incidence. (F) ROC curve analysis of nomogram. The vertical coordinate is true positive, the horizontal coordinate is false positive, and the area below the curve in the figure was called area under the curve (AUC) value. (G) Decision curve analysis (DCA) analysis of nomogram. Different curves represented nomogram, age, risk score.

We categorized the primary clinical features (age, gender, and KPS) and the ultimate survival status of GBM patients in the training set. Following this categorization, we conducted a comprehensive comparative analysis of risk scores across these distinct clinical groups to elucidate potential associations between clinical characteristics and risk stratification. The results revealed a statistically significant difference in risk score solely between age groups (elderly and non-elderly) (p*=*0.005) (Figure 5A). Afterwards, univariate Cox analysis was conducted on the clinical characteristics (age, gender, and KPS) and risk score of the GBM patients in the training set to establish a more accurate prognostic model. The results showed that only the age [HR=1.03 (95 % confidence interval [CI]:1.01–1.04), p<0.001] and risk score [HR=4.9 (95 % CI:2.57–9.34), p<0.0001] were significantly associated with survival (HR≠1, p*<*0.05) (Figure 5B). An independent prognostic component was identified by further multivariate Cox analysis, and the risk score was validated as a risk factor [HR=4.03 (95 % CI:1.77–9.2), p<0.001] (Figure 5C). Finally, to create a nomogram that predicts the 1-, 2-, and 3-years survival rates of GBM patients, age and risk score were chosen as independent prognostic factors (Figure 5D). Calibration curve revealed an acceptable agreement between predicted and actual outcomes, supporting the reliable performance of the nomogram (Figure 5E). The nomogram model’s AUC values at years 1-, 2-, and 3-years were roughly 0.7, according to the ROC curve, which was comparable to the risk score’s AUC values. After adding age diagnosis, the effect was not improved, indicating that the risk score achieved a superior diagnostic effect in comparison to age (Figure 5F). The DCA results illustrated that the combination of risk score and age yielded favorable net clinical benefits across most threshold probabilities (Figure 5G). Collectively, these findings suggest that the risk score possesses stable predictive ability for GBM patients.

### GSEA analysis of prognostic genes and risk score

**Figure 6:**
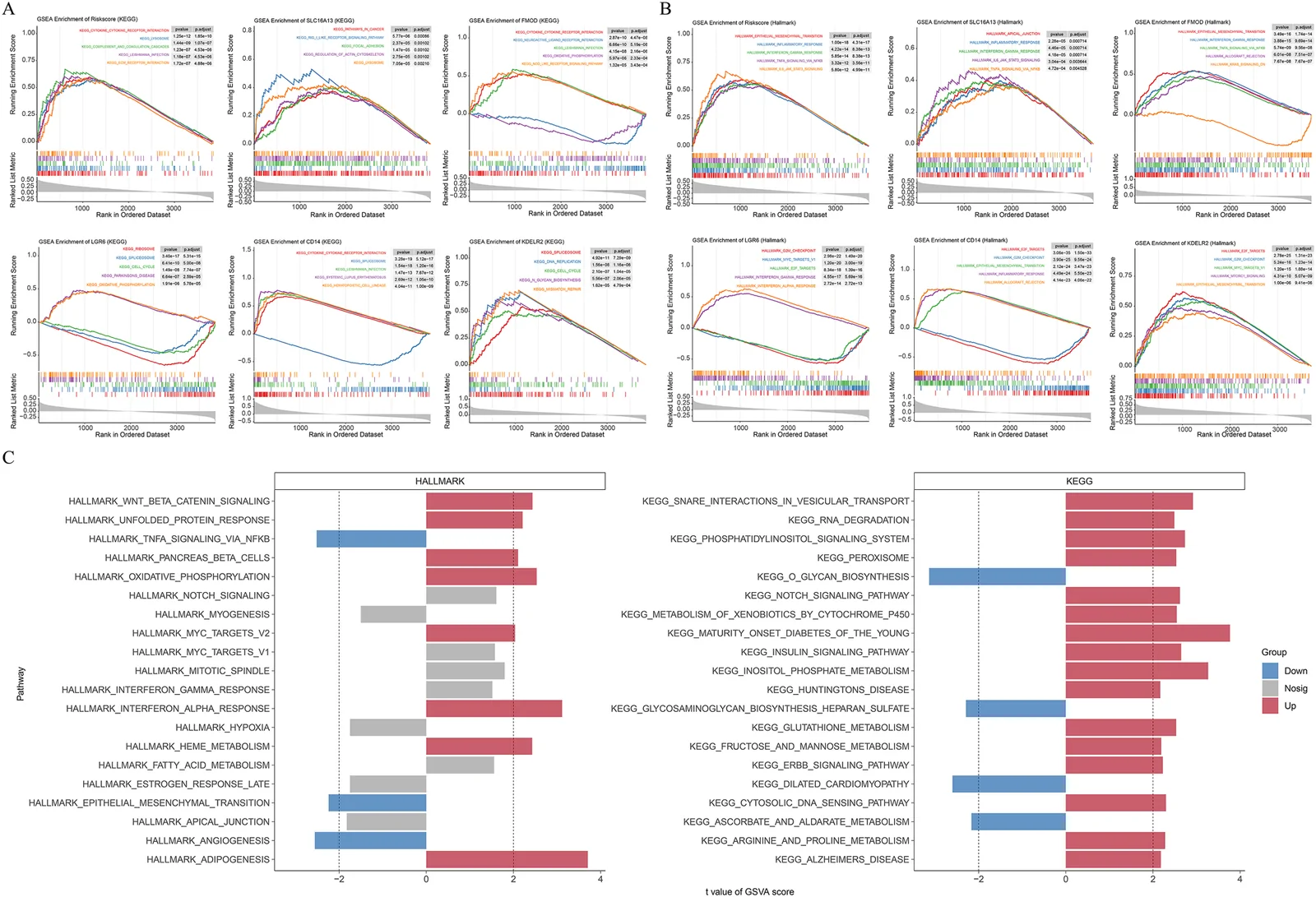
Gene set enrichment analysis (GSEA) and gene set variation analysis (GSVA). (A) GSEA analysis of risk score and prognostic genes (KEGG). (B) GSEA analysis of risk score and prognostic genes (Hallmark). (C) GSVA enrichment analysis in high risk group and low risk group of GBM.

GSEA pathway enrichment analysis was conducted for the 5 prognostic genes and risk score based on KEGG and Hallmark databases, respectively (|NES|>1, p*<*0.05). The results indicated that they were primarily enriched in the pathways related to the cell cycle, G2M checkpoint, epithelial-mesenchymal transition (EMT), and regulation of actin cytoskeleton (Figure 6A and B, Supplementary Tables 5 and 6).

### GSVA analysis between high and low risk groups of GBM

To gain a more profound insight into the variations in pathways between the different risk groups of GBM, we conducted GSVA enrichment analysis (|t|>2, p*<*0.05) (Supplementary Table 7). The top 20 differential pathways in KEGG and Hallmark background gene sets were presented based on the absolute value of t value, as shown from large to small in Figure 6C. The findings showed that, in comparison to the low risk group, multiple pathways were elevated in the high risk group. These pathways including Wnt/β-catenin signaling, unfolded protein response, snare interactions in the vesicular transport, RNA degradation (Figure 6C).

### Immune infiltration and drug susceptibility analyses between high and low risk groups for GBM

**Figure 7:**
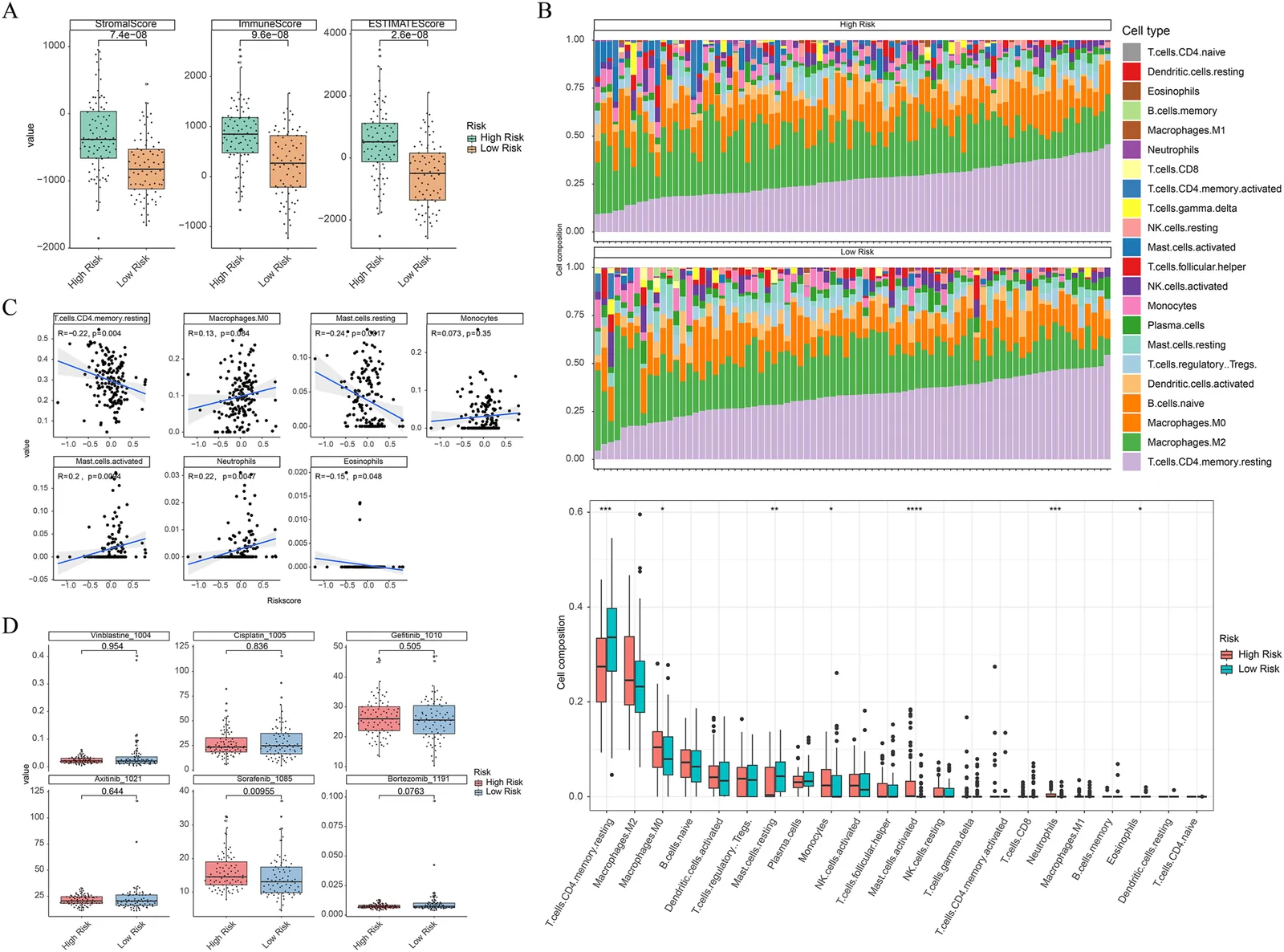
Immune infiltration analysis and drug susceptibility analysis between high and low risk groups for GBM. (A) The immune score, stromal score, and estimate score were analyzed between high and low risk groups. ns indicated p>0.05, *indicated p*<*0.05, **indicated p*<*0.01, ***indicated p*<*0.001, and **** means p*<*0.0001. (B) Analysis of proportion accumulation map and box plot of immune infiltrating cells. ns indicated p>0.05, *indicated p*<*0.05, **indicated p*<*0.01, ***indicated p*<*0.001, and ****means p*<*0.0001. (C) Scatter plot analysis of risk score and immune cells. The horizontal axis was the risk score, and the vertical axis was the degree of immune cells infiltration. (D) IC50 box plot analysis of commonly utilized chemotherapy drugs between high and low risk groups of GBM. ns indicated p>0.05, *indicated p*<*0.05, **indicated p*<*0.01, ***indicated p*<*0.001, and ****means p*<*0.0001.

Significant differences in the tumor microenvironment were observed between the high- and low-risk GBM groups. The StromalScore, ImmuneScore, and ESTIMATEScore all showed markedly higher values in one group over the other, with robust statistical significance (p<0.00001, respectively) (Figure 7A). The percentage abundance bar graphs as well as the percentage box plot of immune cells in the high and low risk group revealed that there were notable variations, including 7 types immune cells, namely resting memory CD4^+^ T cells (p<0.001), M0 macrophages (p<0.05), resting mast cells (p<0.01), monocytes (p<0.05), activated mast cells (p<0.0001), neutrophils (p<0.001), and eosinophils (p<0.05) (Figure 7B, Supplementary Table 8). Based on Pearson correlation analysis between immune cell infiltration levels and risk scores, scatter plots were generated for seven immune cell types. The results revealed statistically significant negative correlations for resting memory CD4+ T cells (R = −0.22, p=0.004), resting mast cells (R = −0.24, p=0.0017), and eosinophils (R = −0.15, p=0.048). Conversely, significant positive correlations were observed for activated mast cells (R=0.20, p=0.0044) and neutrophils (R=0.22, p=0.0047) (Figure 7C, Supplementary Table 9). Although M0 macrophages showed a positive trend (R=0.13), this correlation did not reach statistical significance (p=0.084). Similarly, monocytes demonstrated a non-significant positive association (R=0.073, p=0.35). Drug sensitivity analysis revealed that among the six commonly utilized chemotherapy drugs, sorafenib exhibited a significant difference in IC_50_ values between the different risk groups of GBM (p<0.01) (Supplementary Table 10). The IC_50_ values in the low risk group were notably lower than those in the high risk group, indicating that sorafenib demonstrated higher drug sensitivity in the low risk group (Figure 7D).

### Validation of the prognostic genes

**Figure 8:**
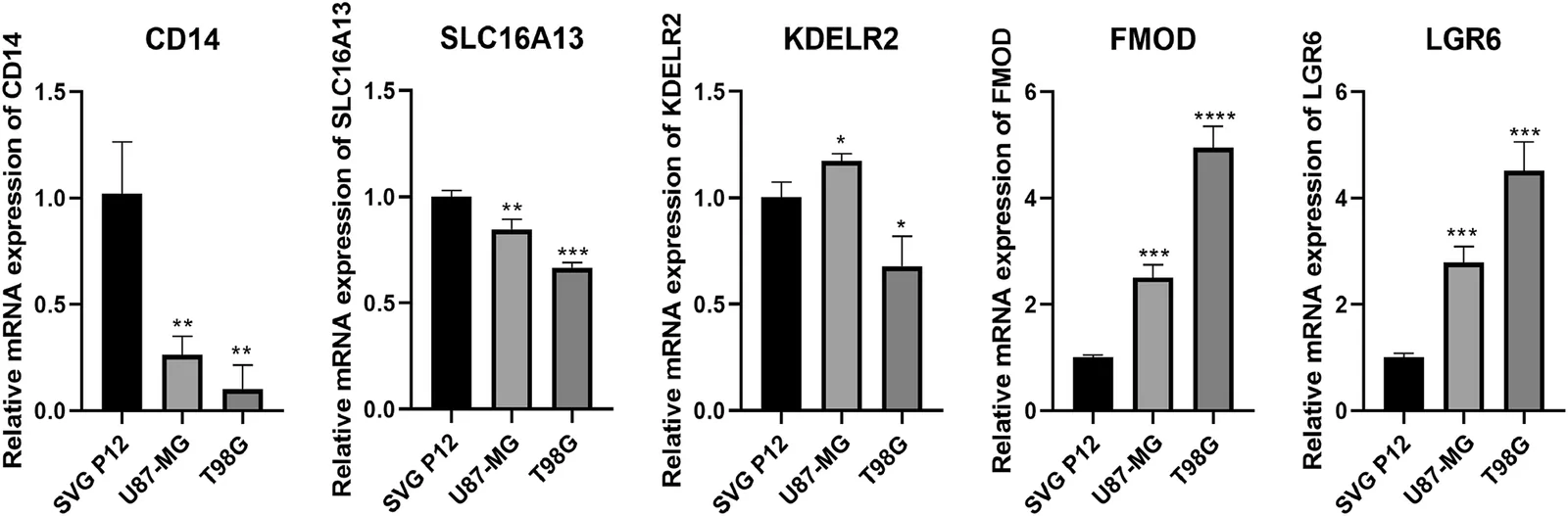
Expression of prognostic genes in normal and GBM cell lines. *Indicated p*<*0.05, **indicated p*<*0.01, ***indicated p*<*0.001, and ****means p*<*0.0001.

To evaluate the robustness of prognostic genes, we purchased the normal cell line (SVG P12) and GBM cell lines (U87-MG and T98G) to verify their expression levels. The results showed that CD14 and SLC16A13 were significantly downregulated in both GBM cell lines, while FMOD and LGR6 were markedly upregulated (Figure 8). The expression pattern of KDELR2 was inconsistent between the two GBM cell lines, which requires further validation.

## Discussion

In the present study, we demonstrated that Golgi function-related genes were closely correlated with GBM prognosis, and a total of 5 pivotal prognostic genes (CD14, FMOD, LGR6, SLC16A13, and KDELR2) were identified through rigorous screening of the TCGA-GBM dataset. Recognized as a key player in inflammatory diseases and tumors [37], Human cluster of differentiation antigen 14 (CD14) expression is elevated in high-grade GBM, where it is primarily localized to tumor cells and infiltrating macrophages/microglia. Through direct interactions with T cells and B cells, CD14 contributes to immunodepletion in the GBM microenvironment [37], and its expression levels can distinguish between different GBM grades [38]. As a co-receptor of the TLR4 complex, CD14 enhances the recognition of inflammatory ligands such as LPS, potentially sustaining a pro-inflammatory tumor microenvironment in GBM [39], [40], [41]. We hypothesize that Golgi glycosylation dysfunction in GBM (evidenced by upregulated GOLPH3 expression and SCAP/SREBP-1 pathway activation [42], 43]) may alter the glycosylation and subsequent membrane localization of CD14, leading to aberrant receptor signaling that could further drive GBM proliferation and invasion. As a member of the leucine-rich small proteoglycan family, Fibromodulin (FMOD) has been reported to be highly expressed in GBM tissues and has been implicated in promoting tumor cell invasion and migration [44]. Notably, FMOD secreted by differentiated glioma cells (DGCs) has been reported to promote angiogenesis by activating integrin-dependent Notch signaling in endothelial cells. However, the underlying mechanisms through which FMOD influences GBM pathogenesis remain incompletely understood and warrant further investigation. Leucine-rich repeat-containing G protein-coupled receptor 6 (LGR6) has been found to be overexpressed in GBM cell lines relative to normal glial cell lines and has been implicated in promoting the malignancy and chemoresistance of GBM, highlighting its potential as a therapeutic target [45]. Within the R-spondin (RSPO) family of secretory proteins – which are processed through the endoplasmic reticulum and Golgi – RSPO2 has emerged as a potent modulator of Wnt signaling. LGR6 expression is known to vary with cellular Wnt activation status, and exogenous enhancement of Wnt pathway signaling upregulates LGR6 levels [46], 47]. The observed upregulation of LGR6 in GBM cell lines suggests its potential role in amplifying tumor proliferation via the Wnt/RSPO signaling axis. Solute carrier family 16 member 13 (SLC16A13), a member of the SLC16A family, functions as a proton-linked monocarboxylate transporter (MCT13) that facilitates the rapid transmembrane movement of metabolites such as lactate. GBM cells exhibit a strong metabolic reliance on lactate shuttling, where lactate and its transporters play key roles in supporting proliferation and migration [48]. While SLC16A13 has been proposed as a possible predictive biomarker for pancreatic cancer [49], its role in GBM remains less explored. We hypothesize that elevated SLC16A13 expression may help maintain an acidic Golgi environment, thereby further promoting rapid tumor proliferation and invasion in GBM. KDEL endoplasmic reticulum protein retention receptors(KDELRs) are proteins that possess 7 transmembrane structural domains, which are crucial for their function in retrieving soluble endoplasmic reticulum-resident proteins from the Golgi complex. Among them, KDELR2 is known to be located in the endoplasmic reticulum-Golgi pathway. By activating the endoplasmic membrane signaling cascade, it enhances Golgi-mediated secretion (especially matrix metalloproteinase MMP), thereby promotes extracellular matrix degradation, facilitating cancer invasion and metastasis [50], 51]. Consistent with these findings, our validation confirms that KDELR2 is highly expressed in GBM tissues and serves as a poor prognostic factor, promoting GBM progression [52], 53]. In conclusion, these GBM-associated Golgi-functional regulators are not merely isolated prognostic markers but collectively contribute to tumor progression by coordinately disrupting core Golgi activities, including glycosylation, secretory trafficking, and ER-Golgi cycling, thereby synergistically enhancing GBM proliferation and invasion.

To evaluate the clinical predictive value of the candidate genes in GBM, we constructed a prognostic model and plotted time-dependent ROC curves. Across multiple datasets, the model yielded stable AUC values of approximately 0.7 at the 2- and 3-year time points, confirming basic prognostic discrimination for preliminary assessment of GBM patient survival. Combined with clinical characteristics, Cox regression was used to screen independent prognostic factors, and both age and risk score were incorporated into nomogram construction. The verification results showed that the AUC of the nomogram model integrating clinical indicators remained at around 0.7, suggesting that its predictive efficacy was at a medium level. It is worth noting that although incorporating age as a factor in the risk scoring did not significantly enhance the predictive power of the model, its evaluation results were superior to those using only the single indicator of age. In conclusion, this GARG prognostic model has certain clinical reference significance, but due to its moderate discrimination efficacy, it is necessary to include a larger sample size, multi-center clinical data, and more clinical characteristics for external validation in the future, and further optimize the model structure to improve its predictive stability and clinical applicability.

We used GSEA analysis to evaluate the biological roles of prognostic genes. The analyses indicated that these prognostic genes were associated with key cellular processes such as the cell cycle, G2M checkpoint, EMT, and regulation of actin cytoskeleton pathway. Studies have shown that the type II transmembrane protein Golgi membrane protein 1 (GOLM1) is located in the cis- and medial-Golgi. It has been confirmed that GOLM1 silencing inhibits GBM cell growth and cell cycle progression, it had been discovered that the Wnt/β-catenin signaling pathway prevents cell invasion and migration [54]. Zhang B et al. demonstrated that the G2M checkpoint pathway is activated in high risk GBM [55]. They also found that targeting the G2M checkpoint can effectively prevent mitosis of cancer cells, thereby exerting control over the proliferation of cancer cells [56]. EMT is a pivotal process that is essential for the migration and invasion capabilities of cancer cells [57]. In addition to the intrinsic biological pathways related to tumor progression, the tumor microenvironment and immune cell infiltration also profoundly shape the malignant progression and clinical outcome of GBM. Therefore, we further explored the differences in the immune microenvironment between high-risk and low-risk subgroups to clarify the immune regulatory characteristics underlying our prognostic model. Resting memory CD4^+^ T cells, M0 macrophages, resting mast cells, monocytes, activated mast cells, neutrophils, and eosinophils exhibited a significant difference in infiltration between the different risk groups of GBM. Among these, M0 macrophages have the ability to be polarized into either M1 or M2 types in response to environmental signals [58]. M1 macrophages are capable of producing pro-inflammatory cytokines and exerting a tumor-killing effect in GBM [59], whereas the M2 macrophage phenotype is closely associated with tumor cell proliferation and poor clinical prognosis in GBM patients [60]. The Golgi apparatus is indispensable for macrophage differentiation, whose morphology and function are regulated by the RhoA signaling pathway. Moreover, macrophages at different polarization states (M0, M1, M2) exhibit distinct dependencies on Golgi-associated signaling [61]. During the development of GBM, eosinophils are activated by GBM mediators, leading to the production of pro-tumor growth factors [62]. Increased infiltration of resting memory CD4+ T cells is linked to poor survival in glioma patients [63], while altered mast cell abundance, monocyte recruitment, and elevated neutrophil-related indicators also serve as important prognostic indicators for GBM [64], [65], [66], [67]. To summarize, these differential immune cell infiltrations closely correlate with the risk stratification of GBM, underscoring their critical role in shaping the tumor’s malignant biological behavior and clinical prognosis.

Considering the clinical application value of this signature, we subsequently evaluated the differences in targeted drug sensitivity among GBM patients with distinct risk levels. Results revealed that the low-risk group had a significantly lower IC50 of sorafenib and was more sensitive to this agent, whereas the high-risk group exhibited sorafenib resist. After reviewing the literature, we found that the association between the resistance of sorafenib in hepatocellular carcinoma (HCC) and the abnormal function of the Golgi apparatus has been confirmed by multiple studies. Specifically, Golgi protein 73 (GP73) is one of the current research hotspots, and it is closely related to the occurrence and development of HCC. GP73 overexpression weakens the sensitivity of HCC cells to sorafenib, and this process is related to the EMT process, which may be one of the important mechanisms by which HCC develops resistance to sorafenib [68]. Another study has shown that extracellular GP73 can work in synergy with alpha-fetoprotein (AFP) to inhibit the anti-tumor effect of sorafenib, thereby enhancing the resistance of HCC cells [69]. In addition, Golgi phosphoprotein 3 (GOLPH3), as another key Golgi-related cancer protein, can mediate sorafenib resistance through exosomes when it is highly expressed in HCC. Mechanistically, GOLPH3 can upregulate the expression of miR-494-3p in exosomes, and this exosomal miR-494-3p can directly target and inhibit the PTEN gene, ultimately promoting angiogenesis in vascular endothelial cells and enhancing the resistance of HCC cells to sorafenib [70]. Although the mechanism of sorafenib resistance in GBM has not been systematically elucidated, the above research results in the field of HCC provide us with important references and preliminary predictions. Based on this, in the future, we will further conduct *in vitro* and *in vivo* experiments to deeply verify the specific roles of GARGs and the signaling pathways they mediate in sorafenib resistance in GBM, in order to provide more direct experimental evidence for individualized clinical treatment of GBM.

Golgi dysfunction is involved in fundamental molecular and cellular biological processes of tumorigenesis, whereas it has been less studied in GBM. To address this gap, our study employed bioinformatics approaches to identify prognostic genes associated with the Golgi in GBM and explored their potential molecular mechanisms, aiming to provide candidate targets for future therapeutic development. With the rapid development of artificial intelligence (including AlphaFold and large biomedical language models) [71], 72], data-driven bioinformatics has become a powerful tool for analyzing the functions of organelles and tumor heterogeneity. This study can provide reference ideas and research directions for subsequent related explorations. In the future, the Golgi-related gene sets and risk stratification system obtained through this study can be further combined with artificial intelligence algorithms, multi-dimensional clinical information, and multi-omics data, which may provide potential research basis for constructing a more accurate GBM diagnosis and prognosis evaluation system and optimizing targeted treatment strategies. However, it is important to acknowledge the limitations inherent in a purely computational methodology. First, the retrospective design of public database-based analyses inevitably introduces selection and temporal biases. Meanwhile, GBM is characterized by prominent intratumoral heterogeneity, which can lead to heterogeneous gene expression profiles across tumor regions and subtly affect the stability of molecular signatures. In addition, several unmeasured clinical confounders, including individualized treatment regimens and MGMT promoter methylation status, could not be fully adjusted in the current cohort. These unstandardized variables may collectively impose moderate impacts on model robustness. As such, the prognostic performance of this risk signature may vary among external cohorts with different clinical characteristics and molecular distributions, which may restrict its broad generalizability to a certain extent. Second, although the established prognostic model can effectively predict sorafenib sensitivity, it cannot stratify the chemotherapeutic response to first-line standard agents for GBM, such as temozolomide. This limitation needs to be further investigated in future studies. Third, while our bioinformatics analyses reveal moderate statistical associations and mechanistic hypotheses, they cannot confirm causal relationships or direct biological functions. The prognostic model and underlying mechanistic findings in this study await further experimental validation, including molecular biology assays for gene expression and functional verification, as well as *in vivo* animal experiments that clarify the exact roles of these GARGs in GBM progression.

## Conclusions

In this study, we successfully identified 5 prognostic genes (CD14, FMOD, LGR6, SLC16A13 and KDELR2) associated with the Golgi in GBM based on bioinformatics analysis, and constructed a prognostic model to forecast the prognosis of GBM patients. These prognostic genes may be associated with the growth and proliferation and also play a crucial role in the migration and invasion of GBM tumor cells. our analyses can provide a better understanding of the pathogenesis of GBM and provide a theoretical basis for early diagnosis and treatment.

## Supplementary Material

Supplementary Material

Supplementary Material

Supplementary Material

Supplementary Material

Supplementary Material

Supplementary Material

Supplementary Material

Supplementary Material

Supplementary Material

Supplementary Material

Supplementary Material
