# Supplementary material for: A novel prognostic model based on Golgi apparatus-related genes predicts patient survival in glioblastoma
Source: Open Med (Wars). 2026 Sep 8;21(1):20261479. doi: 10.1515/med-2026-1479 (PMC13552814; doi:10.1515/med-2026-1479)
Supplement: Supplementary file 1 [file j_med-2026-1479_suppl_001.docx]

**Supplementary information.**

**Supplementary table 1**. Lists for differentially expressed genes (DEGs) in GBM training cohort (TCGA-GBM vs. GTEx normal samples).

**Supplementary table 2.** Functional enrichment analysis of differentially expressed Golgi apparatus-related genes (DE-GARGs).

**Supplementary table 3.** Lists for 65 candidate prognostic genes screened by univariate Cox regression and proportional hazards (PH) assumption testing.

**Supplementary table 4.** Five prognostic genes identified by Lasso-Cox regression analysis with corresponding coefficients.

**Supplementary table 5.** Gene Set Enrichment Analysis (GSEA) results for prognostic genes (KEGG gene sets).

**Supplementary table 6.** Gene Set Enrichment Analysis (GSEA) results for prognostic genes (Hallmark gene sets).

**Supplementary table 7.** Gene Set Variation Analysis (GSVA) of differentially activated pathways between high- and low-risk GBM groups.

**Supplementary tables 8.** Immune cell infiltration profiles quantified by CIBERSORT analysis in high- and low-risk GBM groups.

**Supplementary tables 9.** Correlation analysis between significantly differential immune cells and risk scores.

**Supplementary tables 10.** Drug sensitivity analysis (IC50 values) of common chemotherapeutic agents in high- and low-risk GBM groups.
